# Melatonin to Rescue the Aged Heart: Antiarrhythmic and Antioxidant Benefits

**DOI:** 10.1155/2021/8876792

**Published:** 2021-03-13

**Authors:** Margarita Segovia-Roldan, Emiliano Raúl Diez, Esther Pueyo

**Affiliations:** ^1^Biomedical Signal Interpretation and Computational Simulation (BSICoS), I3A, Universidad de Zaragoza, IIS Aragón and CIBER-BBN, Spain; ^2^CONICET, IMBECU, National University of Cuyo, Argentina

## Abstract

Aging comes with gradual loss of functions that increase the vulnerability to disease, senescence, and death. The mechanisms underlying these processes are linked to a prolonged imbalance between damage and repair. Damaging mechanisms include oxidative stress, mitochondrial dysfunction, chronodisruption, inflammation, and telomere attrition, as well as genetic and epigenetic alterations. Several endogenous tissue repairing mechanisms also decrease. These alterations associated with aging affect the entire organism. The most devastating manifestations involve the cardiovascular system and may lead to lethal cardiac arrhythmias. Together with structural remodeling, electrophysiological and intercellular communication alterations during aging predispose to arrhythmic events. Despite the knowledge on repairing mechanisms in the cardiovascular system, effective antiaging strategies able to reduce the risk of arrhythmias are still missing. Melatonin is a promising therapeutic candidate due to its pleiotropic actions. This indoleamine regulates chronobiology and endocrine physiology. Of relevance, melatonin is an antiaging, antioxidant, antiapoptotic, antiarrhythmic, immunomodulatory, and antiproliferative molecule. This review focuses on the protective effects of melatonin on age-induced cardiac functional and structural alterations, potentially becoming a new fountain of youth for the heart.

## 1. Introduction

Cardiovascular diseases are the first cause of death globally [1, 2], with predominance in older populations. As many different mechanisms are involved in the relationship between cardiovascular disease and aging, a wide range of study models exploring different pathways have been developed to understand these processes [3]. An important proportion of deaths in patients suffering from cardiovascular diseases is associated with abnormalities in the rhythm of the heart, so-called cardiac arrhythmias. Not only in diseased patients but also in healthy populations, age is a major risk factor for arrhythmia development, causing substantial increases in the levels of morbidity and mortality [4, 5]. Due to the extensive structural and functional remodeling of cardiac tissue in the elderly, arrhythmias can relatively frequently occur in individuals who never developed any cardiac condition before. Thus, there is an intensive effort to understand the mechanisms underlying age-induced arrhythmias, from the molecular to the whole-organ level, as well as a great interest in creating new strategies for their prevention that might also ameliorate cardiac function in the elderly.

Despite differences between individuals and species due to molecular mechanisms involving genetic and environmental variability, damage caused by oxidative stress is considered one of the main elements underlying the aging process [6, 7]. Mitochondria have been shown as one of the most important regulators of aging [8]. Reactive oxidative species (ROS), among other oxidative molecules, have been associated with damaged and aged myocardium, playing an important role in cardiac physiology and pathophysiology, as in the case of atrial fibrillation (AF), the most commonly diagnosed cardiac arrhythmia with particularly high prevalence in the elderly [9].

Different drugs have been studied and used in pharmacotherapy to improve the fight against aging and cardiac arrhythmias [10–12]. Although these antiarrhythmic drugs must be used with caution in patients due to their potential adverse effects, positive results of some of them have been reported [13–15], including those of the molecule melatonin. Thanks to its pleiotropic action, melatonin is still considered one of the potentially good candidates for arrhythmia treatment and prevention [16, 17]. Moreover, its antioxidant role should be considered as an important factor to understand its benefits in relation to aging [18–20].

In this review article, we present an overview of arrhythmias and their molecular mechanisms in aged hearts. Considering the pleiotropic effects of melatonin, we focus our interest on the action of this molecule in cardiac tissue. We highlight its potential as a drug against oxidative stress and for antiarrhythmic treatment in aged hearts, which could make melatonin a prospective candidate to become the fountain of youth for the heart.

## 2. Aging: Body and Heart Rhythms

### 2.1. Aging of the Heart

Aging is a complex process. As many human functions decline with age, the incidence and prevalence of diseases increase. Nevertheless, the apparent cycle of life postpones its ending due to human interventions. Improvements in access to shelter, water, and food have doubled life expectancy at birth in the past century. Despite disparities in social support or medical care underlying uneven life expectancy across countries, the proportion of older individuals keeps on increasing globally. This aging of the population comes associated with growing costs related to health care to the elderly all over the world [21].

The relationship between aging and age-related disease remains controversial [21–23]. Some postulate that such a distinction between pathological and nonpathological aging does not invalidate the fundamental underlying molecular mechanisms, which would be potentially shared in the two cases. On the other hand, others support a clear difference between aging and diseases, claiming that aging is not a disease itself but leads to higher vulnerability, implying that different molecular pathways would be involved in each case. For the purpose of this review, we accept that the basic mechanism of aging is the prolonged imbalance between damage and repair [24–26].

Overall, there are three great classes of mechanisms underlying the aging of the organism: (1) inadequate repair of damage, (2) cell number deregulation, and (3) damage caused by oxidative stress [27, 28]. The factors underlying the damage-repair imbalance vary between species and between different individuals of the same species due to the effect of genetic processes and environmental variability.

Specifically in the cardiovascular system, a major problem in aged hearts is the generation of cardiac arrhythmias [29]. Although arrhythmias are most frequently related to heart diseases, they can also occur in nondiseased hearts, with the probability of occurrence exponentially increasing with age. Arrhythmias originating both in the atria and in the ventricles, such as AF, atrial flutter, premature ventricular contractions, ventricular tachycardias, or ventricular fibrillation [30], have an increased prevalence in elderly individuals. Changes in cardiac structure and function that accompany aging and possibly predispose the aged hearts to increased arrhythmic risk have been reported mainly in animal models due to the difficulties in assessing the effects of aging independently of disease in human hearts.

### 2.2. Structural, Electrical, and Autonomic Changes in Aged Hearts

#### 2.2.1. Structural and Autonomic Changes

The heart undergoes age-induced structural changes. Different studies have documented atrial remodeling associated with larger atrial size and volume [31]. In the ventricle, structural remodeling has been associated with left ventricular hypertrophy related to increased wall thickness [32]. Also, there is a loss of cardiomyocytes in the heart by necrotic and apoptotic cell death with age [33]. Cellular mechanisms of senescence are yet to be fully understood in aging. There is evidence about the link between AF and the presence of senescence markers, one of them being p16 (p16^INK4a^, cyclin-dependent kinase inhibitor 2A), which is involved in inflammation. Importantly, aging is characterized by notably increased fibrosis due to a rise in the number of cardiac fibroblasts [34, 35], which are the cells primarily responsible for the production of extracellular matrix components like collagen [36] and proteoglycans [37]. These changes are accompanied by alterations in the cell-to-cell coupling between neighbouring myocytes, which are mediated by changes in gap junction distribution and connexin expression [38].

Aging is additionally associated with changes in the autonomic nervous system (ANS), which regulates the activity of internal body organs like the heart, as well as in the cardiac response to autonomic stimuli [39, 40]. Regarding the sympathetic branch of the ANS, reduced responsiveness to sympathetic nerve stimulation of both aged atria and aged ventricles has been evidenced, although some studies have postulated that this occurs through different mechanisms.Whereas in the aged atria the involved mechanism may be related to decreased *β*-adrenergic responsiveness, in the aged ventricles it may be related to nerve degeneration [41, 42]. Other studies have described that age alters cardiac adrenergic responsiveness, being the age-induced changes related to a reduction in the density of *β*-adrenoceptors and reduced cAMP in association with abnormalities in G protein and kinase activity. Although these changes vary between tissues and between species [43], the ability of the heart to properly respond to autonomic stimuli has been generally documented to decrease with aging [44]. Reduced *β*-adrenoceptor responses and receptor densities have been identified in the aging myocardium [43, 45, 46], exacerbated by decreased cAMP production. It is not fully clear how impaired cAMP-dependent regulation of the slow delayed rectifier potassium current (IKs) [47] and L-type calcium current (ICa-L) [48] translates into alterations of repolarization reserve in the elderly. On the other hand, less research has been dedicated to the investigation of aging effects on the cardiac response to parasympathetic stimuli [49, 50]. Nevertheless, available studies suggest a reduction in parasympathetic regulation with age accompanied by blunted responsiveness of the heart to parasympathetic withdrawal [51]. The density and function of atrial muscarinic type 2 receptors are thought to decline with age [42]. Furthermore, some studies have specifically reported on how age alters the response of cardiac tissue to the interaction between sympathetic and parasympathetic stimuli, showing that the action of *β*-adrenergic stimulation to antagonize cholinergic effects declines in old atria, which could increase arrhythmia susceptibility [52].

The aged-induced structural remodeling and the impaired response to autonomic stimulation in aged hearts, together with the electrical remodeling described in Section 2.2.2, all contribute to reduced cardiac adaptive responses and increased risk for arrhythmias.

#### 2.2.2. Electrophysiological Changes

Cardiac contraction is triggered by a bioelectrical signal, known as action potential (AP). The AP is the result of the electrical potential difference between the intra- and extracellular spaces and involves the opening and closing of complex transmembrane proteins known as ion channels, among other molecules. Both AP generation and propagation are key to ensure proper heart electrical function leading to optimal cardiac output [53, 54].

Aging is associated with remarkable electrical alterations leading to remodeling of both atrial and ventricular tissues [55, 56]. In atrial myocardium, contradictory results on the effects of age on ionic currents have been described, with some studies indicating relevant changes in sodium currents and other studies showing no significant alterations in these currents with age [57, 58]. Increased potassium and decreased calcium currents with age have been observed in atrial myocytes from older animals too. In the ventricular myocardium, decreased potassium currents as well as delayed inactivation and/or increased magnitude of calcium currents, accompanied by adjustments in calcium cycling, have been shown to contribute to prolonged AP with age [59, 60].

In addition to the remodeling leading to delayed and impaired cardiac impulse conduction due to a decrease in Connexin 43 (Cx43) expression with aging [5, 61, 62], there are also electrophysiological alterations in the sinoatrial node involving depression of its activity, which could enhance initiation of the electrical impulse in other cells of the heart [63]. Animal studies suggest that decreased expression of potassium/sodium hyperpolarization-activated cyclic nucleotide-gated channel 2, ICa-L current, T-type calcium current, and voltage-gated potassium (Kv) channels (Kv1.5 channels) can also play a role [64–66].

Thus, age-associated ion channel remodeling in different cardiac regions involves alterations in several families of ion channels, with an impact on the AP [67, 68]. Those channels could be potential targets to help to maintain cardiac function in aged hearts [56].

### 2.3. Mechanisms Underlying Arrhythmias in Aged Hearts

Although evidence suggests that cardiac aging is associated with enhanced predisposition to arrhythmia development, improved knowledge of the mechanisms underlying the enhanced incidence of arrhythmias with aging is still needed. New pharmacological approaches that selectively target the molecular pathways involved in age-induced alterations increasing arrhythmic risk might help prevent cardiovascular diseases and retard the age-related decline in physiological processes.

Age-induced changes in major ionic currents leading to AP disturbances, together with other elements of structural, electrical, and autonomic remodeling in the heart, can increase arrhythmic risk [69, 70]. In particular, an increase in the activity of the sodium-calcium exchanger (NCX) may induce calcium overload, which can trigger arrhythmias [71]. Increased expression of NCX and delayed inactivation of ICa-L, together with reduced expression of SERCA and other proteins related to calcium handling, all contribute to impaired calcium homeostasis and provide mechanisms for increased triggered activity in the aged myocardium [72–76]. In addition, fibrosis accumulation and changes in gap junction distribution and connexin expression with aging lead to slower conduction in cardiac tissue, which increases arrhythmia predisposition [77–82].

Dysfunction or senescence of cardiomyocytes has been observed in aged hearts, with senescent cardiomyocytes exhibiting DNA damage response, endoplasmic reticulum stress, mitochondria dysfunction, contractile dysfunction, hypertrophic growth, and senescence-associated secretory phenotype [83]. Cellular senescence contributes to the loss of function and regenerative capacity of cardiac tissue and, although the environment of an individual determines the degree to which it can be a major causal factor for the organism's functional decline with aging, these changes and damage in the genome can induce inflammation and promote the adverse remodeling described above [84, 85].

**Figure 1:**
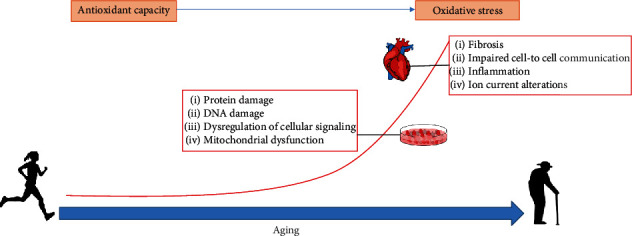
Schematic representation of different mechanisms, including increased production of reactive oxygen species, involved in age-induced remodeling of the heart, from cell to organ level.

Oxidative stress is one of the main damaging processes underlying aging. Indeed, increased production of ROS has been shown to underlie some of the already described elements of age-induced cardiac remodeling (Figure 1). The signaling pathway driven by ROS regulates cardiac development and maturation as well as calcium handling, excitation-contraction coupling, and vascular tone [86, 87]. Excessive ROS production can increase the threshold to trigger cardioprotection and, in relation to the described structural, autonomic, and electrical remodeling, could help explain the increased incidence of damage and the consequent increase in arrhythmia predisposition with aging [5], as further described in Section 3.

## 3. Oxidative Mechanism of Arrhythmias and Antioxidant Antiarrhythmic Therapies

The exposure to different types of damage over time is highly individual specific, partly explaining differences in the aging rate between individuals [88, 89]. Of the many factors contributing to aging of the body, and in particular of the heart, including genetics, epigenetics, diet, smoking, physical activity, and even chance [90–92], here, we will focus on the relationship between oxidative stress and age-induced arrhythmias and we will revise antioxidant therapies that could provide antiarrhythmic benefits.

### 3.1. Oxidative Stress in the Aging Heart and Relation to Arrhythmic Risk

Although the mechanisms responsible for senescence and remodeling in cardiomyocytes and aged hearts are not fully understood, mitochondria have emerged as central regulators of the aging process [8, 86]. In the aged myocardium, mitochondrial function is impaired at various levels including ROS formation, dynamics, and metabolism. Together with ROS, which is considered to be one of the primary determinants of aging [93–95], the superoxide anion, the hydroxyl radical, and hydrogen peroxide are in fact crucial molecules with important roles in cardiac physiology and pathophysiology through various signaling pathways [96].

Oxidative stress can work as a mediator of AF, with elevated levels of ROS having been linked to it [97, 98], both oxidative stress and AF being common in aged hearts. Also, prooxidant genes are overexpressed and many antioxidant genes are downregulated in AF. In addition, derivatives of reactive oxidative metabolites and ratios of oxidized to reduced glutathione and cysteine show high levels in blood from patients suffering AF [99]. ROS facilitates ventricular arrhythmia too, especially in aged and hypertensive rat hearts, with underlying mechanisms involving the generation of early afterdepolarizations [100].

Among the various possible ways for ROS to facilitate arrhythmia development, alterations in ionic currents have been revealed as an important factor [7, 101–103]. Ionic alterations due to elevated ROS have been observed in the sodium-potassium ATPase (Na/K-ATPase) pump, NCX, and K^+^ and Na^+^ channels [104, 105]. In addition, the molecular mechanism of arrhythmias induced by ROS could be linked to hydrogen peroxide (H_2_O_2_), a major contributor to oxidative stress, producing complex cardiac effects that may involve altered calcium homeostasis [106]. Moreover, there is evidence about how ROS may act on the ICa-L current producing a leak of Ca^2+^ from the sarcoplasmic reticulum (SR). Besides, ROS decreases peak sodium current and SERCA-mediated SR Ca^2+^ uptake. ROS participates in the inhibition of KATP channels [107] and decrease of the transient outward potassium current (Ito) [108], among others [109]. All these mechanisms are related to an increase in intracellular Ca^2+^ levels, AP prolongation, reduction in conduction velocity, and facilitation of triggered activity and reentry. Also, dysregulation of L-type calcium channels and an increase in late Na^+^ current may result in arrhythmia [110–112]. The decrease in total Na^+^ current induced by elevated ROS may cause a reduction in conduction velocity and provide a substrate for reentry [113].

On top of oxidative stress causing alterations in ion channels, its potential contribution to cardiac arrhythmias by impairing intercellular communication has been additionally described, both in relation to aging and to various pathological states. This effect may result in changes in conduction properties. Specifically, elevated ROS levels decrease the expression and distribution of Cx43, the main component of gap junction channels in the ventricles and one of the most important connexins in the atria [7, 114, 115]. This impairs conduction through gap junctions and may facilitate cardiac arrhythmias [116, 117]. Also, the effect of ROS radicals on cardiac structure by promoting high levels of fibrosis may impede the normal propagation of the AP across the heart. This effect is due to proliferation of cardiac fibroblasts by the high level of transforming growth factor beta 1 (TGF-*β*1) production induced by increased ROS [118].

On the basis of the described mechanisms underpinning oxidative stress as a key contributor to arrhythmias, further studies delving into these different routes could help to identify molecules that can serve as therapeutic targets for oxidative stress in the aged heart with antiarrhythmic benefits.

### 3.2. Markers of Oxidative Stress: Role on Antiarrhythmic Therapies

Increased serum markers of oxidative stress might be an important guide in selecting the individuals who will most likely respond to antioxidant therapy and could benefit from its possible antiarrhythmic effects [99, 119]. Studies exploring novel molecular mechanisms for ROS-induced arrhythmia have led to the discovery of new potential antiarrhythmic targets.

Among the mentioned potential mechanisms to be considered as targets against arrhythmias via oxidative stress therapies, one of them is the abnormal splicing of cardiac sodium channel mRNA in heart failure. This is a possible mechanism underlying decreased sodium current in ranges associated with sudden cardiac death, suggesting that interruption of this processing could potentially reduce the arrhythmic risk [113, 120]. Moreover, Ca^2+^/calmodulin-dependent kinase II (CaMKII) can be activated by ROS and its activation likely mediates several of the reported ROS-induced arrhythmogenic effects [121–123]. Several studies have recently described the link between the use of CaMKII inhibitors and the improvement in catecholaminergic polymorphic ventricular tachycardia, helping to inhibit arrhythmic phenotypes [124]. As an additional mechanism that could represent a potential source of new antiarrhythmic targets, it is worth mentioning the impact of oxidative stress on the increased activity of ryanodine receptors (RyRs). Protein kinase A hyperphosphorylates cardiac RyRs, which results in a dysregulation of SR Ca^2+^, provoking a leak of calcium in failing hearts that can trigger fatal ventricular arrhythmias [10, 125–132].

However, the role of ROS scavengers is hampered by the ability of ROS radicals to react with other molecules. A general scavenger can usually neutralize one or a few forms of ROS, but other forms of ROS remain to exert their proarrhythmic effects. Clinical trials using general antioxidants have not shown the expected therapeutic outcome. A more effective antioxidant therapeutic approach targeting the sources of ROS could be more beneficial. Among cardiac sources of ROS, mitochondria and NADPH oxidase seem to be promising therapeutic targets for the management of AF, a common arrhythmia in old age [133, 134]. Because mitochondria play a central role in ROS-induced ROS release, mitochondria-targeted antioxidant therapy may prove to be the most effective antioxidant therapy for the management of chronic AF [86, 135–137].

The free radical theory of aging attributes aging processes to the accumulation of damage inflicted by free radical species. Among the different species involved in aging, we should mention some antioxidants such as superoxide dismutase, catalase, and metallothionein [138]. In Section 4, we will focus our attention on the role of melatonin as a potential antioxidant molecule against aging.

## 4. Melatonin as a Potential Antiarrhythmic Treatment in Aging

### 4.1. Melatonin and Cardiac Activity

Even if further studies in humans are needed, melatonin appears as a key molecule in heart aging. Melatonin is an amphipathic and pleiotropic molecule, being able to act on several targets at different levels and cell locations [139, 140]. Melatonin was first discovered in the 1950s by Lerner et al. [141], and it is considered the molecule of the night, due to its increased levels during nighttime in the majority of animal species. Melatonin is mainly secreted by the pineal gland [142], and its production is considered to follow a rhythmical pattern, making it a molecule involved in biological seasonal and yearly rhythms of the organisms [143].

Melatonin has a double role in the sleep-wake cycle. This molecule is able to entrain and shift the circadian rhythm (chronobiotic function) and, in addition, plays its action on the sleeping process, helping through a “hypnotic” function by increasing the homeostatic drive to sleep [144]. At the level of the pineal gland, production and release of melatonin follow a clear circadian pattern, having its peak level of synthesis during nighttime [145]. Melatonin has been reported to have a cardioprotective role in various animal species [146]. In fact, increases in melatonin during nighttime are linked to a good cardiovascular function in different organisms. Reduced levels of melatonin are related to disease, aging, and chronodisruption.

Melatonin has an apparent antioxidant function, slows down aging, and expands the life expectancy, helping to delay or avoid different age-related diseases [147, 148]. The molecular mechanisms underlying those processes are still not fully elucidated. The role of melatonin as a cardioprotective molecule has been suggested as part of a pathway linked with its antioxidative action in the heart [149]. However, other mechanisms related to ion channel interaction as well as positive effects in mitochondria define its cardioprotective action too. Melatonin acts via its receptors, which are expressed throughout the body, including cardiac tissue [18]. The pleiotropic action of this molecule in cardiac tissue makes melatonin an interesting agent in preventive treatments, particularly in relation to cardiac arrhythmias. Since the work by Tan et al. in 1998 [150], our understanding of melatonin as an antiarrhythmic molecule has increased significantly, becoming an important and promising cardioprotective molecule for the heart. Arrhythmia incidence has been documented to decrease during nighttime hours, when melatonin levels increase 30- to 70-fold. In particular, life-threatening cardiac arrhythmias (ventricular tachycardia, ventricular fibrillation, and sudden cardiac death) are more likely to occur in the morning after waking up, when melatonin levels are lower or even undetectable.

**Figure 2:**
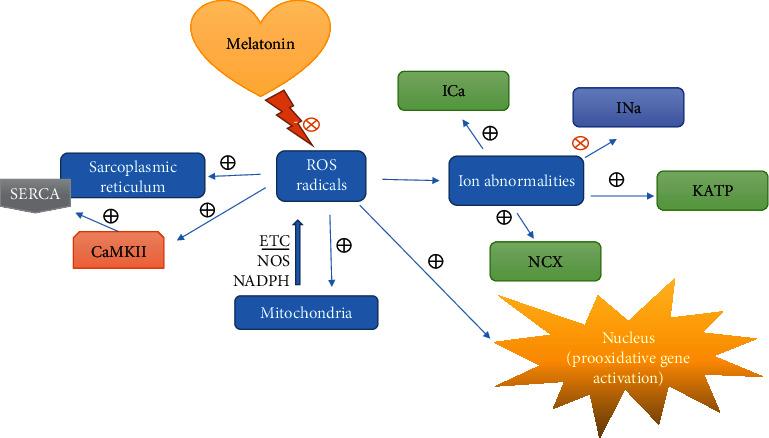
Scheme about the effect of reactive oxygen species (ROS) in cardiac tissue at different levels. Also, the potential role of melatonin as an antioxidant molecule and its implications for arrhythmia prevention are illustrated (CaMKII: Ca^2+^/calmodulin-dependent kinase II; SERCA: sarco/endoplasmic reticulum Ca^2+^-ATPase; ROS: reactive oxygen species; ICa: calcium current; INa: sodium current; KATP: ATP-sensitive potassium channel; NCX: sodium-calcium exchanger; ETC: electron transport chain; NOS: nitric oxide synthases; NADPH: nicotinamide adenine dinucleotide phosphate hydrogen).

In summary, melatonin presents different mechanisms of action involving receptor activation, ion channel modulation, improvement of mitochondrial functions, antioxidant effects, and others. Based on these mechanisms and their implications to the aging process, melatonin is considered a key antiaging element through several pathways (Figure 2).

### 4.2. Melatonin Receptors

Melatonin receptors are characterized to have a wide range of locations. They can be found in different regions within the cell, the plasma membrane being a relevant location linked to the interaction with the melatonin molecule. Melatonin receptors can be found in several systems and organs like the nervous system, kidneys, liver, intestinal tract, prostate gland, uterus, skin, and eyes, as well as the heart and the cardiovascular system [151]. These receptors, named MT1 and MT2, and also known as Mel1a and Mel1b, can play their role on melatonin action in the organisms via cell membrane interaction and following a pathway involving G proteins [152]. This melatonin interaction with its receptors at the level of the plasma membrane occurs with high affinity between the molecule of melatonin (Kd ~ 0.1 pM) and each of MT1 and MT2 [153, 154].

Regarding the structure of the melatonin receptor, both MT1 and MT2 can develop their role as monomers and dimers. The MT1 homodimer forms in a proportion 3- to 4-fold higher than the MT2 homodimer and the MT1/MT2 heterodimer. In addition, there is an MT3 receptor, described as a quinone reductase 2, with an affinity in the nanomolar ranges [155]. Both MT1 and MT2 inhibit adenylate cyclase-protein kinase A-CREB signaling in target cells by pertussis toxin-sensitive G*α*i, *β*, and *γ* and toxin-insensitive Gq, *β*, and *γ* proteins [151, 156]. The MT1 receptor also increases phosphorylation of mitogen-activated protein kinase 1/2 (MAPK) and extracellular signal-regulated kinase 1/2 (ERK) and increases the potassium conductance through inwardly rectifying (Kir3.x) channels. The effect on potassium channels could be relevant to heart electrophysiology since Kir3.x channels are highly expressed in cardiomyocytes and are usually coupled to acetylcholine and adenosine membrane receptors [157]. In the case of the MT2 melatonin receptor, its activation inhibits both forskolin-stimulated cAMP production and cGMP formation, activates protein kinase C (PKC) in the nervous system, and decreases calcium-dependent dopamine release in the retina. Native functional MT1/MT2 heterodimers in mouse rod photoreceptors mediate melatonin's enhancement of scotopic light sensitivity through phospholipase C and PKC pathways [158]. Different drugs such as Luzindole work as receptor blockers, with an important role in cardiac tissue. Luzindole and 4P-PDOT competitively block MT1 melatonin receptors (in concentrations higher than 300 nM), and both are inverse agonists in systems with constitutively active MT1 receptors [148–155, 159].

Melatonin can act in the regulation of calcium levels due to its interaction with intracellular proteins such as calmodulin, calreticulin, or tubulin [160], all of these pathways being potentially linked to the action of melatonin in the cardiovascular system directly or indirectly [161]. In particular, the role of the molecule of melatonin in mitochondrial pathways has been described. Melatonin provides a potent protection against mitochondrial-mediated lesions, playing an important role in the organism in terms of energy production [162].

### 4.3. Antioxidant Effects of Melatonin

Deficiencies in the electron transport chain from mitochondria generates reactive oxygen species and reactive nitrogen species (ROS/RNS) [163–167]. Oxidative stress decreases respiratory complex activity, impairs the electron transport system, and opens the mitochondrial permeability transition pores leading to cell death [165–169]. Melatonin has an antioxidant effect through mitochondria, improving and preserving the good function of complexes I and III and preventing the opening of the mitochondrial permeability transition pores [170–173]. In addition, melatonin prevents the high activity of NADPH oxidase, which can be linked to the production of oxidative species beyond mitochondria, considered as the main source of oxidizing species during oxidative phosphorylation [18, 164, 174].

The reduction of mitochondrial damage in the heart could be related to the negative regulation of angiotensin II type 1 receptor (AT1) by melatonin [13]. In addition, sirtuin-1 and sirtuin-3 are downstream mediators of the cardioprotective actions of melatonin. Sirtuin-3 is a family member that is primarily located in the mitochondria and protects against inflammation and diseases related to oxidative stress. Melatonin elevates sirtuin-3, stimulates superoxide dismutase activity, and suppresses mitochondrial oxidative stress and, via sirtuin-1 and sirtuin-3, it can also prevent isoproterenol-induced mitochondrial injury [175].

Following the previously described pathways, melatonin has been described to have an effect as a powerful antioxidant, with a potency up to 10 times greater than vitamin E [176], having an important role interacting against oxidative species. Free radicals and nonradical reactive species have been documented as one of the main molecules involved in aging processes, and melatonin could play its role against them [163, 177]. Redox biology involves the study of ROS, RNS, and reactive sulfur species as well as their role in the damage of several molecules and tissues. Under physiological conditions, ROS/RNS act as second intracellular messengers modulating signal transduction pathways [164]. A good balance between the production and the removal of free radicals is key to maintaining proper concentrations of this species in the organism, thus avoiding damage. Melatonin may play a crucial role in preserving such a good balance. However, when oxidants increase above healthy levels, oxidative stress occurs and represents a severe risk to the molecular integrity of lipids, proteins, and DNA. Different molecules, including melatonin, could work in the neutralization of reactive species as scavenger molecules, generating a chemical way of counteracting oxidative stress. In particular, the role and interaction with mitochondria could be a good strategic point, as this organelle is considered the main source of oxidation. However, as mentioned before, there are many other pathways to be detected, which create other oxidant species, such as NADPH oxidase, which have their origin in a different system beyond the mitochondrial one.

It is possible to classify different types of antioxidants as (1) nonenzymatic ones (melatonin, vitamins, and GSH) and (2) enzymatic ones (SOD, catalase, thioredoxin, and glutathione peroxidase). It is worthy to mention that the melatonin molecule is 5 times more effective than GSH, for instance [167, 178]. Melatonin stimulates antioxidant enzymes by acting on membrane, cytoplasmic, and nuclear receptors [178, 179]. Low melatonin concentrations increase the expression or activity of SOD, catalase, and glutathione peroxidase [178, 180]. In addition, melatonin can prevent oxidative stress. The aromatic indole ring of melatonin reduces and repairs electrophilic radicals acting as an electron donor. One molecule of melatonin can neutralize up to 10 toxic reagents, including ROS, RNS, and other free radicals. Moreover, several metabolites formed when melatonin neutralizes harmful reagents are also antioxidants, suggesting that a cascade of reactions increases the efficacy of melatonin. Being a highly lipophilic and hydrophilic compound, melatonin crosses all morphological barriers and acts not only in each cell but also within each subcellular compartment. Additionally, melatonin increases the efficacy of vitamin E, vitamin C, and GSH. Therefore, the elimination of free radicals can be carried out by intracellular interactions independent of any receptor [18].

Since free radicals are continuously produced from oxidative reactions and melatonin production declines through the human lifespan, exogenous melatonin could be used in the aging population to improve health and reduce free radical damage.

### 4.4. Cardioprotective Effects of Melatonin

The beneficial effect of melatonin on health, disease, and aging has been supported by many in vivo animal studies. In particular, the potential of melatonin intake to improve cardiac function and diminish oxidative stress has been demonstrated [181]. The antiarrhythmic effects of melatonin were first attributed to its notable antioxidant properties. Melatonin has been later suggested as a good candidate for cardiovascular diseases and aging due to its role in modulating electrophysiological properties from cell to tissue levels, its relation to oxidative stress, and its important action in repairing and controlling circadian rhythms [182]. Loss of the circadian rhythm regulated by melatonin predisposes the heart to suffer cardiac arrhythmias, particularly ventricular tachycardia, due to conduction disorders and changes in repolarization [15]. Since melatonin levels decline progressively over a lifespan [171, 183], loss of melatonin during aging could contribute to a gradually increased predisposition to hypertension and arrhythmias [18].

Melatonin has been shown to have a cardioprotective role, including an antiarrhythmic effect, through different pathways. Melatonin prevents renal damage and arrhythmogenic myocardial remodeling during unilateral ureteral obstruction due to a decrease in oxidative stress, fibrosis, and apoptosis associated with AT1 reduction and Hsp70-VDR increase [13]. Other studies have reported a low level in the synthesis of melatonin and low circulating levels of this molecule in patients with coronary heart disease as well as in patients with hypertension and heart failure [184–190]. Cardiac events, such as arrhythmias [191] and others [192], more likely occur during the morning hours when levels of melatonin are considered to be lower [193]. This evidence supports the crucial role of endogenous melatonin in cardiovascular pathologies [194]. Melatonin can have an antiapoptotic effect associated with restoring Nrf2 antioxidant capacity (one of the main molecules responsible for detecting oxidative stress damage), improving this way mitochondria ultrastructure altered by aging [195]. Additionally, cardioprotection by melatonin against oxidative stress and arrhythmias could be widely explained by modulation of ion channels, AP and Cx43 expression [196–198].

The action of melatonin in the opening of Kir3.x channels could explain its control of membrane potential [199]. In the nervous system, there is an effect of melatonin via Kir.3x channels and linked to the circadian rhythm [200]. In fact, it is possible to see an effect on circadian sleep regulation through suppression of GABAergic neurons by melatonin in the lateral hypothalamus (a crucial function for wakefulness) through Kir3.1/Kir3.2 channels and via the MT1 receptor [199]. This interaction via the MT1 receptor inactivates hyperpolarization-activated cyclic nucleotide-gated channels [201]. Due to their important role in the nervous system, Kir channels can also be involved in the heart rate decrease mediated by the parasympathetic branch of the autonomic nervous system [202].

Regarding sodium channels (Na^+^), oxidative stress can increase the late sodium current through direct Na^+^ channel modification, prolonging the AP and facilitating the generation of arrhythmogenic triggers known as early afterdepolarizations (EADs) [203–205]. In the case of calcium channels (Ca^2+^), a redox regulation can occur in cardiac myocytes via ryanodine receptors, IP3 receptor and ICa-L [105, 206]. Different species such as ROS and RNS affect the L-type Ca^2+^ channel Cav1.2 through cysteine residues that can alter the molecular levels of intracellular Ca^2+^ or modify specific signaling molecules as in smooth muscle [207]. It has been reported how a low-affinity interaction between melatonin and calmodulin antagonizes the binding of Ca^2+^, involved in its antioxidant action and in electrophysiological signaling [208–211]. In addition, melatonin acutely increases ICa-L in chick cardiac membranes [212] and downregulates voltage-sensitive calcium channels in the rat heart [213].

Voltage-gated potassium (Kv) channels contributing to myocardial repolarization are sensitive to oxidative stress [135, 214]. Particularly, the sulfenic acid, a conserved cysteine residue of Kv1.5 channels, can induce arrhythmia under prolonged oxidative stress [18, 214, 215]. The role of melatonin may depend on time and doses of administration, as it happens via contact with certain channels such as Kv1.3 in lymphocytes or other mechanisms in cardiomyocytes [216]. An example of a dose-dependent action of melatonin is the interaction with KATP channels via mitochondria. The preventive action of melatonin on the permeability transition pore in this channel has been described as an important part of its neuroprotective effect [217]. Its role against mitochondrial dysfunction has been described on myocardial tissue too [170–173]. Nevertheless, the effect in the opening of KATP channels in cardiac tissue at high concentrations of melatonin could be proarrhythmic [218, 219].

On top of the important role of melatonin as a modulator of ion channel properties, it has also a crucial and positive effect in conduction in cardiac tissue due to its interaction with Cx43, the most abundant connexin in the heart [220]. Connexin proteins assemble into intercellular channels at gap junctions. The largest accumulation of connexins occurs in specialized structures at the ends of cardiomyocytes called intercalated discs. The lateral borders of the myocytes usually show variable amounts of gap junctions depending on age or disease. Melatonin receptor activation has been shown to protect against low potassium-induced ventricular fibrillation by shortening the AP, preserving ventricular electrical activation, and preventing acute changes in Cx43 distribution [221]. Melatonin has been shown to prevent myocardial abnormalities arising due to structural changes and modification of connexin localization and to improve cardiac conduction [198]. Additionally, melatonin has been shown to protect against arrhythmias by increasing the myocardial Cx43 by PKC in hypertensive rats [198].

Although most studies on melatonin action in the heart focus on its antiarrhythmic protection in relation to its antioxidant properties [222–228], new antiarrhythmic effects have been associated with oxidative stress-independent action on other cardiac properties, such as ventricular activation [197], thus opening the route for the study of additional attributes of this promising therapeutic agent. Some studies have documented an antiarrhythmic effect of melatonin in isolated hearts of female rats when administered continuously from the stage prior to the onset of myocardial ischemia [196]. Also, melatonin reduces arrhythmias when administered during reperfusion. Specifically, melatonin has shown to have a protective action when administered to isolated hearts of hypertensive rats [14]. These animals show greater activity of the enzyme NADPH oxidase, which is one of the main systems for generating free radicals, and, therefore, higher levels of oxidative stress, which is involved in the generation of early and delayed afterdepolarizations [229]. The acute administration of melatonin during low potassium perfusion reduced the incidence of ventricular fibrillation and improved the recovery of sinus rhythm. Protection was mediated by activation of melatonin receptors and by prevention of dephosphorylation and lateralization of Cx43 [221].

### 4.5. Pharmacological Consideration Regarding Melatonin Administration

Melatonin is a safe molecule without serious adverse effects in a broad range of concentrations either when administered orally or intravenously [230–234]. For antiarrhythmic purposes, two main approaches should be taken into account: first, an acute administration in the context of high proarrhythmic conditions or to treat an ongoing arrhythmia, and second, a chronic preventive one for situations with increased risk of arrhythmias.

The intravenous bioavailability of melatonin is 100% after a bolus administration and decays biexponentially with half-lives within 2 minutes and 20 minutes [235]. Oral administration reaches a peak concentration around 45 minutes with bioavailability ranging from 3 to 33%, according to different reports. Due to its hydro- and lipophilic properties, melatonin can easily reach the membranes and the inner organelles. Therefore, the potential as an antiarrhythmic to act in the acute scenario is clearly available. However, the evidence is still mainly preclinical [14, 198, 221, 229, 236]. In the context of ischemia/reperfusion, simultaneous intravenous and intracoronary administration has been proven to be electrophysiologically safe. Formulations of melatonin in polyethylene glycol or saline solution at doses from 14 to 50 mg did not affect the electrophysiological response of the myocardium under ischemia/reperfusion conditions [237, 238]. Unfortunately, both clinical studies failed to prove the cardioprotective effect of melatonin against infarct size. The studies were not designed to evaluate severe reperfusion arrhythmias like sustained ventricular tachycardia and ventricular fibrillation.

Oral doses in an acute cardiac scenario ranged from 3 mg to 25 mg and displayed a safe electrophysiological profile despite mixed cardioprotection results. A conservative approach of 3 mg of melatonin, started on the night following the primary percutaneous coronary intervention and continued daily during the hospitalization, had mixed results on enzymatic myocardial infarct size [239]. Pretreatment with melatonin during five days before coronary artery bypass grafting surgery reduced myocardial injury, heart rate, oxidative stress, and inflammatory markers when compared to placebo [240]. On the other hand, 25 mg melatonin administered for twelve weeks following acute coronary syndrome may aggravate endothelial dysfunction [241]. Interestingly, the combination of 50 mg intravenously and 10 mg orally display cardioprotection in patients undergoing abdominal aortic aneurysm repair, with a risk reduction of 25% of cardiac morbidity, including postoperative atrial fibrillation [242].

Several safety studies showed the lack of severe side effects of melatonin during chronic and sustained administration [243–245]. Regarding arrhythmias, one report of two patients suffering frequent premature ventricular contractions claimed that arrhythmic events resolved after stopping melatonin 1 mg as their sleeping pills [246]. On the other hand, several studies validated the melatonin safety profile during long periods of supervised side effect evaluation. No prospective clinical research has been conducted to evaluate melatonin as an antiarrhythmic. Several preclinical studies postulate melatonin as a preventive agent against ventricular arrhythmias [13, 15, 197, 198, 247, 248]. The proarrhythmic vs. antiarrhythmic effect of melatonin deserves further translational and clinical studies.

## 5. Preventive Perspectives: The Fountain of Youth for the Heart

**Figure 3:**
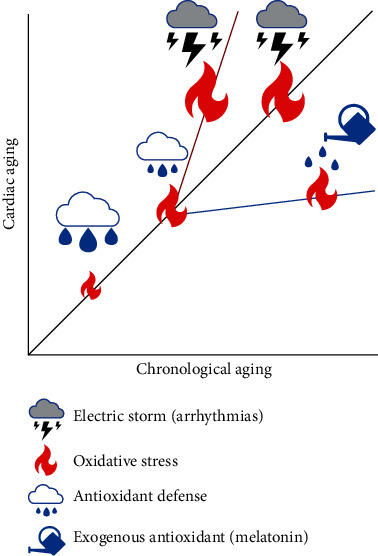
The balance between oxidative stress and antioxidant defenses influence the evolution of cardiac age-induced remodeling and the level of proarrhythmic risk. Increases in oxidative stress anticipate the occurrence of arrhythmic events, whereas antioxidants delay or even abrogate arrhythmias.

We gave an overview of the positive effect of melatonin and focused our attention on its role as a future possible treatment aimed at reducing the risk of cardiac arrhythmias in aged hearts. As described in this review, the structural and functional remodeling that accompanies aging increases susceptibility to atrial and ventricular arrhythmias in humans and in animal models. The increase in oxidative stress with aging plays a major role in this age-associated remodeling. When antioxidant defenses, which decline with aging, are not able to counteract the elevated levels of oxidative stress, arrhythmias can manifest with higher probability. On this basis, we bring here a summary of the variety of positive effects of melatonin. This molecule could be one of the new keys to find the fountain of youth against adverse age-related remodeling of the heart (Figure 3). Several drugs have been described as novel, potential antiarrhythmic agents mainly for the management of AF in the elderly. However, more research is expected to lead to new strategies for prevention or treatment of arrhythmias in the elderly, including ventricular arrhythmias.

Previous studies have described the protective role of melatonin against autophagy and apoptosis in cardiomyocytes via regulation of mitochondrial uncoupling protein 2 (UCP2), producing a mechanism of protection against lipopolysaccharides (LPS) [249]. However, the protective mechanism of melatonin goes far beyond this action, involving different roles in the nervous system [250–252], myocardium remodeling [17] and antioxidant properties [162], all these factors generally involved in its receptor-mediated actions linked to antiarrhythmic properties. This substantiates that melatonin becomes a molecule worthy to be further explored for use in clinical applications. Elevated heterogeneities in cardiac conduction and AP repolarization, which may occur in relation to aging and other cardiac diseases, can be reduced by melatonin via its action in the modulation of myocardial Cx43 [198] and ion channels, improvement of cardiomyocyte physiology, and generation and conduction of APs, ultimately protecting against arrhythmogenesis [18, 56].

Moreover, melatonin synchronizes central but also peripheral oscillators like the heart, allowing temporal organization of biological functions through circadian rhythms and improving adaptation of the individual to the internal and external environment, thus working as a good antiaging agent [182]. Furthermore, numerous studies have confirmed the antiarrhythmic protection of melatonin and have related it to its remarkable antioxidant properties under a time- and dose-dependent response. As an example of these facts, studies in hypertensive rat hearts, which have a higher activity of the enzyme NADPH oxidase implicated in oxidative stress and present high arrhythmic risk, have shown that treatment with melatonin has positive effects, avoiding arrhythmias, either sustained ventricular tachycardia or ventricular fibrillation [18, 198, 229].

Based on the number and diversity of major melatonin benefits, there is also a perspective for measuring melatonin not only as a possible treatment to defeat cardiovascular problems but also as a biomarker to detect, control, and follow-up certain disorders. Melatonin could become essential for clinical preventive and therapeutic applications in newborns, children, and adults based on its physiological regulatory effects [182]. However, it is crucial to take into account that its role can be time- and dose-dependent so as to avoid possible adverse effects [246, 253]. In addition, considering that the ability of the pineal gland to produce melatonin is compromised during aging, with a gradual decrease in blood melatonin levels at night, the potential use of melatonin as an early biomarker indicative of adverse age-induced remodeling associated with arrhythmic risk is anticipated [183].

Among the list of potential antiarrhythmic molecules and based on the antioxidant action and pleiotropic effects of melatonin in organisms, it has been described as one of the potential antioxidant targets in cardiac tissue [226]. Melatonin concentrations and its associated effects can highly vary from one individual to another as well as in relation to aging or chronic diseases. Among many effects, melatonin has shown an important role as an inhibitor of lipid peroxidation. Due to this effect, melatonin plays a crucial role in stabilizing cell and organelle membranes, with the final goal of protecting them. Besides, melatonin can act as a direct free radical scavenger and also an indirect antioxidant. Additionally, its metabolites are efficient in scavenging ROS and reactive nitrogen species. Melatonin also plays an effective action related to mitochondrial homeostasis [165]. Based on cell energy impairment, apoptosis, and overproduction of ROS as pathogenic mechanisms in aging melatonin could play a crucial role in counteracting deleterious aging [135, 137], effects in the cardiovascular system, particularly considering its antioxidant action. In conclusion, melatonin is a molecule representing a new target to be further tested in clinical trials expanding its application beyond sleep problems or a few already explored disorders. Future studies on dose-response relationships will be crucial to extend the use of melatonin in clinical practice, for both treatment and prevention, particularly in the context of cardiac arrhythmias in aged hearts.
